# The *piggyBac* Transposon-Mediated Expression of SV40 T Antigen Efficiently Immortalizes Mouse Embryonic Fibroblasts (MEFs)

**DOI:** 10.1371/journal.pone.0097316

**Published:** 2014-05-20

**Authors:** Ning Wang, Wenwen Zhang, Jing Cui, Hongmei Zhang, Xiang Chen, Ruidong Li, Ningning Wu, Xian Chen, Sheng Wen, Junhui Zhang, Liangjun Yin, Fang Deng, Zhan Liao, Zhonglin Zhang, Qian Zhang, Zhengjian Yan, Wei Liu, Jixing Ye, Youlin Deng, Zhongliang Wang, Min Qiao, Hue H. Luu, Rex C. Haydon, Lewis L. Shi, Houjie Liang, Tong-Chuan He

**Affiliations:** 1 Department of Oncology and Southwest Cancer Center, Southwest Hospital, Third Military Medical University, Chongqing, China; 2 Molecular Oncology Laboratory, Department of Orthopaedic Surgery, The University of Chicago Medical Center, Chicago, Illinois, United States of America; 3 Ministry of Education Key Laboratory of Diagnostic Medicine, and the Affiliated Hospitals of Chongqing Medical University, Chongqing, China; 4 Department of Laboratory Medicine, the Affiliated Hospital, Binzhou Medical University, Yantai, Shandong, China; 5 Department of Cell Biology, Third Military Medical University, Chongqing, China; 6 Department of Orthopaedic Surgery, Xiang-Ya Hospital of Central South University, Changsha, China; 7 Department of Surgery, Zhongnan Hospital of Wuhan University, Wuhan, China; 8 School of Bioengineering, Chongqing University, Chongqing, China; Shanghai Jiao Tong University School of Medicine, China

## Abstract

Mouse embryonic fibroblasts (MEFs) are mesenchymal stem cell (MSC)-like multipotent progenitor cells and can undergo self-renewal and differentiate into to multiple lineages, including bone, cartilage and adipose. Primary MEFs have limited life span in culture, which thus hampers MEFs’ basic research and translational applications. To overcome this challenge, we investigate if piggyBac transposon-mediated expression of SV40 T antigen can effectively immortalize mouse MEFs and that the immortalized MEFs can maintain long-term cell proliferation without compromising their multipotency. Using the *piggyBac* vector MPH86 which expresses SV40 T antigen flanked with flippase (FLP) recognition target (*FRT*) sites, we demonstrate that mouse embryonic fibroblasts (MEFs) can be efficiently immortalized. The immortalized MEFs (piMEFs) exhibit an enhanced proliferative activity and maintain long-term cell proliferation, which can be reversed by FLP recombinase. The piMEFs express most MEF markers and retain multipotency as they can differentiate into osteogenic, chondrogenic and adipogenic lineages upon BMP9 stimulation *in vitro*. Stem cell implantation studies indicate that piMEFs can form bone, cartilage and adipose tissues upon BMP9 stimulation, whereas FLP-mediated removal of SV40 T antigen diminishes the ability of piMEFs to differentiate into these lineages, possibly due to the reduced expansion of progenitor populations. Our results demonstrate that *piggyBac* transposon-mediated expression of SV40 T can effectively immortalize MEFs and that the reversibly immortalized piMEFs not only maintain long-term cell proliferation but also retain their multipotency. Thus, the high transposition efficiency and the potential footprint-free natures may render piggyBac transposition an effective and safe strategy to immortalize progenitor cells isolated from limited tissue supplies, which is essential for basic and translational studies.

## Introduction

Mouse embryonic fibroblasts (MEFs) are multipotent progenitor cells with the capacity of differentiating into tissues of both mesenchymal and non-mesenchymal origin [1]–[6]. MEFs can differentiate into osteoblastic, chondrogenic, and adipogenic lineages [1]–[6], although MEFs are also capable of differentiating into other lineages, such as neuronal [7]–[9] and cardiomyogenic [10] lineages. MEFs have attracted significant attention for their potential role in stem cell biology and regenerative medicine [5], [6], [11]–[15]. MEFs can be isolated from almost every type of tissue, including bone marrow stromal, periosteum, brain, liver, bone marrow, adipose, skeletal muscle, amniotic fluid and hair follicle [5], [6], [11].

One of the major technical challenges is to isolate sufficient MEFs for *in vitro* and *in vivo* studies, as well as to expand MEFs for possible clinical applications [5], [6], [11]. One approach to overcome such challenge is to conditionally or reversibly immortalize MEFs with high efficiency. The classical 3T3 cell immortalization protocol is not efficient [16]. Most recent approaches involves in the stable expression of oncogenes and/or inactivation of tumor suppressor genes [17]. One of the most commonly used immortalizing genes is SV40 T antigen [18]–[20]. We and others previously used retroviral vector-mediated expression of SV40 T antigen to immortalize primary cells [21]–[27]. However, the immortalization efficiency was relatively low, largely due to the low viral titters of large cargo size for retroviral packaging. Thus, the bottleneck of efficient immortalization is to effectively deliver the immortalizing factors into the targeted primary cells.

The *piggyBac* transposon system has emerged as one of the most promising non-viral vector systems for efficient gene transfer into mammalian cells [28]. Transposons are mobile genetic elements that can be used to integrate transgenes into host cell genomes. The *piggyBac* transposon was originally isolated from the cabbage looper moth, Trichoplusiani, and has been recognized as one of the most efficient DNA transposons for manipulating mammalian genomes [28]–[31]. The *piggyBac* transposon system has two major components, a donor plasmid carrying the gene of interest flanked by two terminal repeat domains and a helper plasmid expressing *piggyBac* transposase that catalyzes the movement of the transposon. We engineered the *piggyBac*-based immortalization vector pMPH86, which expresses a drug selection markers and the SV40 T antigen flanked with flippase (FLP) recognition target (FRT) sites, while it remains to be tested how efficiently this vector can immortalize primary cells.

In this study, we sought to investigate 1) if the piggyBac vector pMPH86 can effectively immortalize primary mouse embryonic fibroblasts (MEFs); and 2) if the immortalized MEFs can maintain long-term proliferation without compromising the multipotent differentiation potential. We demonstrate that co-transfection of pMPH86 and piggBac transposase expression vector can effectively immortalize MEFs with an enhanced proliferative activity. The immortalized MEFs (piMEFs) express most of the MSC markers. Using the previously demonstrated potent osteogenic, chondrogenic, and adipogenic factor BMP9 [32]–[36], we demonstrate that BMP9 can up-regulate the key lineage-specific regulators Runx2, Sox9 and PPARγ2, and that BMP9 can induce osteogenic and adipogenic differentiation in vitro. Exogenous FLP expression in piMEFs leads to an effective removal of SV40 large T antigen and results in a significant decrease in cell proliferation. Stem cell implantation studies indicate that the piMEFs can form bone, cartilage and adipose tissues upon BMP9 stimulation, whereas the FLP-mediated removal of SV40 T antigen significantly diminishes the ability of piMEFs to differentiate into these tissues, possibly due to the reduced expansion of progenitor populations. Taken together, our results demonstrate that *piggyBac* transposon mediated expression of SV40 T can effectively immortalize MEFs and that the reversibly immortalized piMEFs not only maintain long-term cell proliferation but also retain the ability to differentiate into multiple lineages. Thus, it is conceivable that the high transposition efficiency and footprintless natures of *piggyBac* transposon may offer an effective and safe strategy to immortalize progenitor cells isolated from limited tissue supplies, which may be critical for basic and translational studies.

## Materials and Methods

### Cell Culture and Chemicals

HEK-293 cells were from ATCC (Manassas, VA) and maintained in the completed Dulbecco's Modified Eagle Medium (DMEM) described [32], [37]–[40]. Unless indicated otherwise, all chemicals were purchased from Sigma-Aldrich or Fisher Scientific.

### Isolation of Mouse Embryo Fibroblasts (MEFs) and Establishment of Immortalized MEFs (piMEFs)

The animal welfare, use, and care were carried out according to the approved protocol by the Institutional Animal Care and Use Committee (IACUC) of The University of Chicago (protocol #71108). MEFs were isolated from post coitus day 12.5–13.5 CD1 mice as described [23], [40]–[42]. Briefly, embryos were dissected into 10 ml sterile PBS, voided of internal organs, and sheared through 18-gauge syringes in the presence of 0.25% trypsin/1 mM EDTA. After 15 min incubation with gentle shaking at 37°C, DMEM with 10% fetal bovine serum (FBS) was added to inactivate trypsin. The cells were plated onto 100 mm cell culture dishes and incubated for 24 h at 37°C. Adherent cells were used as MEF cells. Aliquots were kept in LN2 tanks. All MEFs used in this study were within five passages.

**Figure 1:**
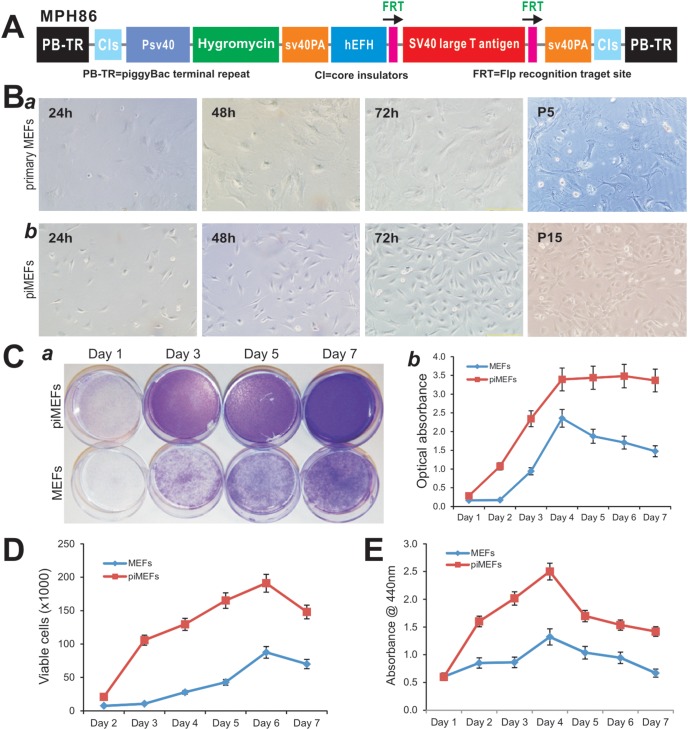
The *piggyBac* transposon-mediated SV40 T expression effectively immortalizes mouse embryonic fibroblasts (piMEFs) and exhibits higher proliferative activity than that of primary MEFs. (**A**) Schematic representation of the *piggyBac* reversible immortalization vector MPH86. This piggyBac vector contains the hygromycin and SV40 T antigen expression cassette flanked by FRT sites. (**B**) Morphology of primary and immortalized mouse embryonic fibroblasts (MEFs). (***a***) Primary MEFs were seeded at 20–30% confluence and passed consecutively for five passages (P5). (***b***) The piMEF cells were seeded at low density and passed consecutively for 15 passages (P15). (**C**) Cell viability and proliferation assay by crystal violet staining assay. The same numbers of primary MEF and piMEF cells were seeded at a low density. Cells were stained with crystal violet at the indicated time points (a) and the viable and stained cells were dissolved for OD reading as described [43], [44] (b). (**D**) Cell counting assay. The same number of primary MEF and piMEF cells was seeded at 20% confluence. Cells were trypsinized, stained with Trypan blue, and counted at the indicated time points. (**E**) Cell proliferation assessed with WST-1 assay. The same cell number of primary MEFs and piMEFs was seeded at a low density. Cell culture medium was collected for WST-1 assay at the indicated time points. For all of the above assays, each assay condition was done in triplicate. The assays were repeated in at least two independent batches. Representative results are shown.

To establish the immortalized MEFs (piMEFs), early passage MEFs (<3 passages) were seeded in 25 cm^2^ flasks and co-transfected with piggyBac vector MPH86, which expresses SV40 T Ag flanked with FLP (**Fig. 1A**), and the piggyBac transposase expression vector, Super PiggyBac (SBI, Mountain View, CA). Stable piMEF cell pools were established by selecting the transfected cells with hygromycin B for one week. Aliquots of the piMEFs were kept in liquid nitrogen tanks.

### Cell Viability/Proliferation Assay (Crystal Violet Assay)

Subconfluent cells were seeded in 12-well plates, infected with the indicated adenoviruses or without any treatments. Cells were subjected to crystal violet staining at the indicated time points. Staining images were taken from the plates. For quantitative measurement, the stained cells were dissolved in 10% acetic acid at room temperature for 20 min with agitation. Absorbance was measured at 570∼590 nm [43], [44].

### Cell Proliferation WST-1 Assay

Exponentially growing cells were plated into 96-well culture plates at 20% confluence. Wells without seeding any cells were used as background controls. At the indicated time points, 4 µl of the premixed WST-1 (BD Clontech, Mountain View, CA) were added to each well and incubated at the 37°C CO_2_ incubator for 1 hr. 100 µl of the WST-1-containing culture medium were taken for absorbance reading at 440 nm using a plate reader. The obtained A_44__nm_ values were subjected to background reading subtractions. Each assay condition was done in triplicate.

### Recombinant Adenoviruses Expressing BMP9, Flippase (FLP), Red Fluorescent Protein (RFP) and Green Fluorescent Protein (GFP)

Recombinant adenoviruses were generated using the AdEasy technology [32], [33], [45], [46]. The coding regions of human BMP9, FLP recombinase and GFP were PCR amplified and subcloned into adenoviral shuttle vector and subsequently used to generate recombinant adenoviruses in HEK293 cells, resulting in adenoviruses AdBMP9 and AdFLP, which also express GFP as a marker for monitoring infection efficiency. Analogous control adenovirus expressing only GFP (AdGFP) or monomeric RFP (AdRFP) was used as a control [44], [47]–[50].

### RNA Isolation and Semi-quantitative RT-PCR (sqPCR) Analysis

Total RNA was isolated using TRIZOL Reagents (Invitrogen) and subjected to reverse transcription reaction with hexamer and M-MuLV Reverse Transcriptase (New England Biolabs, Ipswich, MA). The cDNA products were diluted 10- to 100-fold and used as PCR templates. sqPCR was carried out as described [22], [24], [35], [37], [40], [41], [48], [51]–[53]. PCR primers for mouse Runx2 (5′-CCG GTC TCC TTC CAG GAT-3′ and 5′-GGG AAC TGC TGT GGC TTC-3′; accession no. NM_001145920), Sox9 5′-AGC TCA CCA GAC CCT GAG AA-3′ and 5′-TCC CAG CAA TCG TTA CCT TC-3′; accession no. NM_011448), PPARγ2 5′-ACT GCC GGA TCC ACA AAA-3′ and 5′-TCT CCT TCT CGG CCT GTG-3′; accession no. NM_011146) transcripts were designed by using *Primer3 Plus* program (http://www.bioinformatics.nl/cgi-bin/primer3plus/primer3plus.cgi) to amplify the genes of interest (approximately 150–250 bp). A touchdown PCR program was carried out as follows: 94°C for 2 min for 1 cycle; 92°C for 20 s, 68°C for 30 s, and 72°C for 12 cycles with a decrease in 1°C per cycle; and then at 92°C for 20 s, 57°C for 30 s, and 72°C for 20 s for 20–25 cycles, depending on the transcript abundance. The PCR products were confirmed by resolving PCR products on 1.5% agarose gels. All samples were normalized by the expression level of GAPDH.

### FACS Analysis

Subconfluent cells were harvested, fixed with 70% ethanol, washed with PBS and stained with Hoechst 33342. Cell cycles were analyzed using the BD LSR II Flow Cytometer and the FlowJo software. Each assay condition was done in triplicate.

### Alkaline Phosphatase (ALP) Activity Assay

ALP activity was assessed quantitatively with a modified assay using the Great Escape SEAP Chemiluminescence assay kit (BD Clontech) and qualitatively with histochemical staining assay (using a mixture of 0.1 mg/ml napthol AS-MX phosphate and 0.6 mg/ml Fast Blue BB salt) as described [32], [33], [35], [37], [40]–[42], [51], [54], [55]. Each assay condition was performed in triplicate.

### Matrix Mineralization Assay (Alizarin Red S Staining)

Cells were seeded in 24-well culture plates, infected with AdBMP9 or AdGFP, and maintained in the presence of ascorbic acid (50 µg/mL) and β-glycerophosphate (10 mM). At 14 days post infection, mineralized matrix nodules were stained for calcium precipitation using Alizarin Red S staining as described [32], [33], [35], [37], [40]–[42], [51], [54], [55]. Briefly, cells were fixed with 0.05% (v/v) glutaraldehyde at room temperature for 10 min and washed with distilled water, fixed cells were incubated with 0.4% Alizarin Red S for 5 min, followed by extensive washing with distilled water. The staining of calcium mineral deposits was recorded under a bright field microscope.

### Immunofluorescence Staining

Immunofluorescence staining was performed as described [22], [24], [35], [37], [40], [52]. Briefly, cells were fixed with methanol, permeabilized with 1% NP-40, and blocked with 10% BSA, followed by incubating with CD73, CD44, CD90, CD117/c-kit, CD29, CD133, CD105/endoglin, CD166/ALCAM, or BMPR-II antibody (Santa Cruz Biotechnology) for 1 hr at room temperature. After being washed, cells were incubated with Texas Red-labeled secondary antibody (Santa Cruz Biotechnology) for 30 min. Cell nuclei were stained with DAPI. Stains were examined under a fluorescence microscope. Stains without primary antibodies, or with control IgG, were used as negative controls.

### Subcutaneous Stem Cell Implantation

All animal work was conducted according to the approved protocol by the Institutional Animal Care and Use Committee (IACUC) of The University of Chicago (protocol #71108). Stem cell-mediated ectopic bone formation was done as described [33], [35], [40]–[42], [48], [53]. Briefly, MEFs cells were co-infected with AdBMP9+AdFLP or AdBMP9+AdGFP for 16 h, collected and resuspended in PBS for subcutaneous injection (5×10^6^/injection) into the flanks of athymic nude (nu/nu) mice (5 per group, 4–6 wk old, female, Harlan Laboratories, Indianapolis, IN). At 4 wk after implantation, animals were sacrificed, and the implantation sites were retrieved for µCT imaging, histologic evaluation, and other specialty stains.

### Micro-Computed Tomography (µCT) Analysis

All retrieved specimens were fixed and imaged using the µCT component of the GE triumph (GE healthcare, Piscataway, NJ, USA) trimodality preclinical imaging system. All image data were analyzed with Amira 5.3 (Visage Imaging, Inc., San Diego, CA), and 3D volumetric data were obtained as described [23], [42], [53], [56], [57].

### Hematoxylin & Eosin, Trichrome, and Alcian Blue Staining

Retrieved tissues were fixed in 10% formalin overnight and embedded in paraffin. Serial sections of the embedded specimens were stained with hematoxylin and eosin (H & E). Trichrome and Alcian Blue stains were carried out as described [23], [32], [33], [35], [48], [53].

### Statistical Analysis

All quantitative experiments were performed in triplicate and/or repeated three times. Data were expressed as mean ± SD. Statistical significances were determined by one-way analysis of variance and the student’s *t* test. A value of *p*<0.05 was considered statistically significant.

## Results

### *piggyBac* Transposon-mediated Expression of SV40 T Antigen Effectively Immortalizes Mouse Embryonic Fibroblasts (piMESs), which Exhibit High Proliferative Activity

We previously used the retroviral vector-mediated expression of SV40 T antigen to immortalize several sources of progenitor cells, including MEFs [22]–[27]. However, the immortalization efficiency was relatively low because of the low retrovirus titers associated with the large cargo size for packaging. We thus sought to investigate if piggyBac transposon-mediated expression of SV40 T antigen can effectively immortalize MEFs, while retaining the multipotent properties of MEFs. The engineered piggyBac vector, designated as pMPH86, contains the hygromycin and SV40 T antigen expression cassettes flanked with FRT sites (**Fig. 1A**). Primary MEFs were shown to grow fairly well, albeit at lower rate, up to at least five passages (**Fig. 1B** **, panel a**). The immortalized MEFs (piMEFs) grew more rapidly and maintained a high proliferation rate after 15 passages (**Fig. 1B** **, panel b**). In fact, piMEFs have been passed more than 40 generations thus far, and maintain high proliferative activity, indicating that we successfully immortalized MEFs.

We next compared the proliferative activities between primary MEFs and piMEFs. Crystal violet staining assay indicated that piMEFs reached confluence as early as day 3 while primary MEFs reached confluence at day 7, when both started with a similar cell density (**Fig. 1C** **, panel a**). Quantitative assessment of the stained cells confirmed that piMEFs had significantly higher cell staining at each time points than that of the MEFs’ (**Fig. 1C** **, panel b**). Direct cell counting further revealed that piMEFs grew faster than primary MEFs (**Fig. 1D**). Likewise, the piMEFs exhibited a higher proliferation rate than that of MEFs in the quantitative WST-1 assay when the same number of primary MEF and piMEF cells was seeded at a low density (**Fig. 1E**). Taken together, these results demonstrate that immortalized MEFs, or piMEFs can be maintained in culture and exhibit much higher proliferation rate.

### piMEFs Express MSC Markers

**Figure 2:**
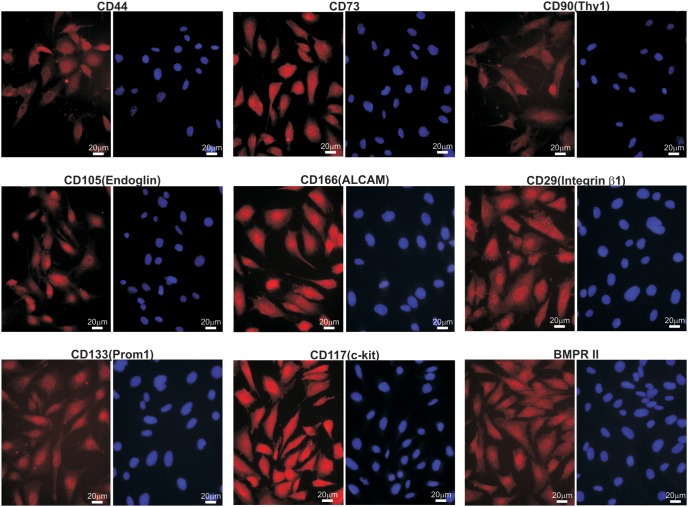
The piMEFs express mesenchymal stem cell markers. The piMEF cells were seeded at subconfluency for 24/Thy-1, CD73, CD105/Endoglin and CD166/ALCAM [58]. Other mesenchymal and/or progenitor markers include CD29/Integrin β1, CD117/c-kit, CD133/Prom1, and BMPR II. Cell nuclei were stained with DAPI. Respective IgG Isotypes were used as immunostaining control.

We examined the expression of MSC markers using immunofluorescence staining in order to verify if the piMEFs retain MSC features. While no single marker can be used to identify MSC cells, the reported consensus MSC markers include CD90/Thy-1, CD73, CD105/Endoglin, CD166/ALCAM and CD44 [58]. We found that all of these markers were readily detectable in piMEFs by immunofluorescence staining (**Fig. 2**). In addition, we found that the piMEFs readily express other MSC and/or progenitor markers, including CD29/Integrin β1, CD133/Prom1, CD117/c-kit, and BMPR II (**Fig. 2**). We did not detect any expression of CD45 and CD34 in the piMEFs (data not shown). Thus, these results demonstrate that piMEFs express most if not all of the common MSC markers, suggesting that they may retain MSC-like phenotypes.

### piMEF Cells are Capable of Differentiating into Osteogenic, Chondrogenic, and Adipogenic Lineages

**Figure 3:**
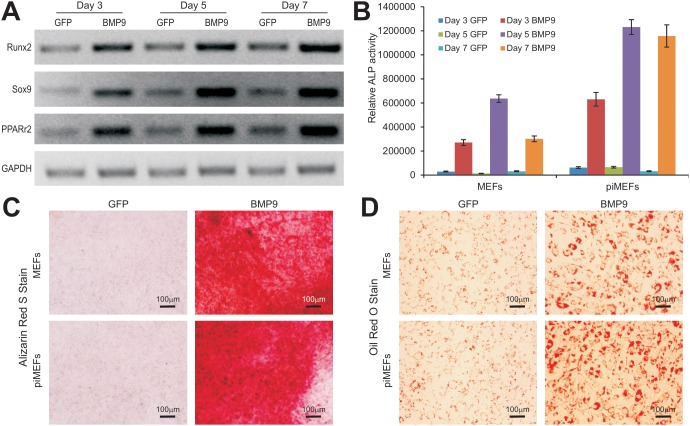
Induction of osteogenic, chondrogenic, and adipogenic lineage markers in piMEFs *in vitro*. (**A**) Expression of lineage-specific regulators in piMEFs stimulated by BMP9. Subconfluent piMEF cells were infected with AdBMP9 or AdGFP. Total RNA was isolated at the indicated time points and subjected to RT-PCR reactions. The cDNA products were used as templates for semi-quantitative amplification of mouse Runx2, Sox9 and PPARγ2 transcripts. All samples were normalized with GAPDH expression levels. (**B**) Induction of early osteogenic marker alkaline phosphatase (ALP) in primary MEFs and piMEFs. Subconfluent primary MEFs and piMEFs were infected with AdBMP9 or AdGFP. ALP activity was quantitatively determined at days 3, 5 and 7. (**C**) Matrix mineralization assessed with Alizarin Red S staining. Subconfluent MEFs and piMEFs were infected with AdBMP9 or AdGFP and maintained in mineralization medium for 14 days. Cells were fixed and stained with Alizarin Red S. (**D**) Adipogenic differentiation assessed with Oil Red O staining. Subconfluent MEFs and piMEFs were infected with AdBMP9 or AdGFP for 10 days. Cells were fixed and stained with Oil Red O staining. Each assay condition was done in triplicate. The assays were repeated in at least two independent batches. Representative results are shown.

We tested if the piMEFs were able to differentiate into the common lineages derived from MEFs. We previously demonstrated that BMP9 is one of the most potent factors that can induce osteogenic and adipogenic, to a lesser extent, chondrogenic differentiation [32]–[36], [59]. When the piMEFs were transduced with AdBMP9 or AdGFP, the three major lineage-specific regulators, Runx2 (osteogenic), Sox9 (chondrogenic) and PPARγ2 (adipogenic), were significantly up-regulated at as early as day 3 (**Fig. 3A**). Moreover, the early osteogenic marker alkaline phosphatase (ALP) was assessed, BMP9 was shown to induce much higher ALP activity in the piMEFs than that in primary MEFs at the tested time points (**Fig. 3B**). The BMP9-transduced piMEFs were further shown to effectively undergo late stage of osteogenic differentiation as evidenced by matrix mineralization assessed with Alizarin Red S staining (**Fig. 3C**). When MEFs and piMEFs were stimulated with BMP9 for 10 days, Oil Red O staining assay revealed that piMEFs can undergo adipogenic differentiation, as efficiently if not more efficiently as that of the primary MEFs (**Fig. 3D**). Alcian Blue staining of the cultured micromass indicated that piMEFs were able to differentiate to chondrogenic lineage upon BMP9 stimulation (data not shown). Thus, these results are consistent with what we previously reported in MEFs [32]–[36], [59]. Thus, these in vitro results demonstrated that the piMEFs are multipotent and capable of differentiating into osteogenic, chondrogenic and adipogenic lineages.

### The Proliferative Activity of the piMEFs can be Reversed by FLP Recombinase

**Figure 4:**
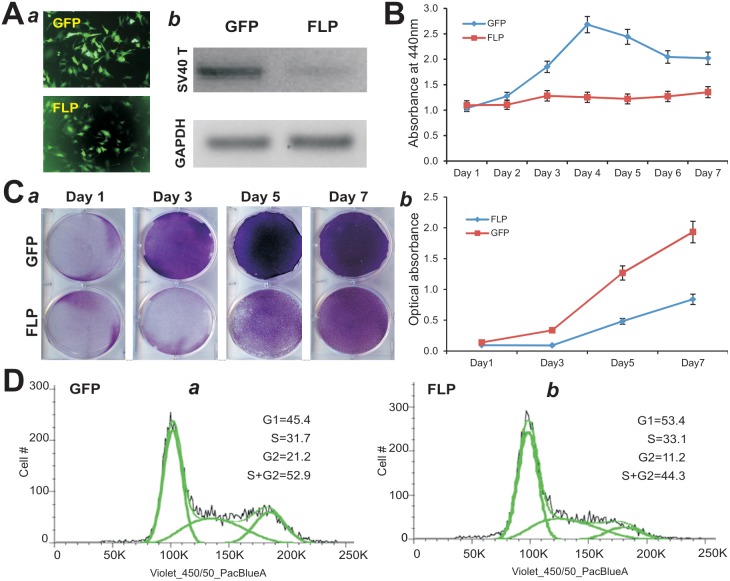
The proliferation properties of the piMEFs can be reversed by FLP. (**A**) Efficient removal of SV40 T antigen from piMEFs by FLP. (***a***) Efficient transduction of piMEFs by adenoviral vectors. Subconfluent piMEFs were infected with AdFLP or AdGFP for 24 h. GFP signal was recorded under fluorescence microscopy. (***b***) FLP-mediated efficient removal of SV40 T antigen detected by qRT-PCR. Subconfluent piMEFs were infected with AdFLP or AdGFP for 36 h. Total RNA was collected and subjected to reverse transcription. The cDNA products were used for semi-quantitative PCR using SV40 T specific primers. The products were resolved on agarose gels. GAPDH served as an internal control. The assays were done in triplicate. (**B**) Cell proliferation WST-1 assay. Subconfluent piMEFs were infected with AdFLP or AdGFP. Cell culture medium was collected for WST-1 assay at the indicated time points. Each assay condition was done in triplicate. (**C**) Cell proliferation assessed by crystal violet staining. AdFLP or AdGFP-transduced piMEFs were stained with crystal violet at indicated time points (***a***), and the stained cells were dissolved for OD reading (***b***). (**D**) Cell cycle analysis. Subconfluent piMEFs were infected with AdFLP or AdGFP. Cells were collected at 48 h post infection and subjected to cell cycle analysis. Each assay condition was done in triplicate. Representative results are shown.

As shown in **Fig. 1A**, the immortalizing gene SV40 large T antigen could be removed through the action of FLP recombinase on the flanking FRT sites. It is conceivable that the immortalization-related phenotypes of the piMEFs could be reversed by FLP recombinase. To effectively express FLP in the piMEFs, we constructed a recombinant adenoviral vector AdFLP, which was shown to transduce piMEFs with high efficiency (**Fig. 4A** **,** panel **a**). The efficient removal of SV40 T antigen by FLP was confirmed by RT-PCR analysis of the SV40 T antigen expression in AdFLP infected piMEFs, but not in the GFP control (**Fig. 4A** **,** panel **b**). Cell proliferation rate of the AdFLP-transduced piMEFs was significantly decreased as assessed by WST-1 proliferation assay (**Fig. 4B**). Similarly, the proliferation and survival of the AdFLP-transduced piMEFs significantly reduced as assessed by Crystal violet staining (**Fig. 4C** panel **a**) and quantitative determination of the stained cells (**Fig. 4C** panel **b**). Quantitative cell cycle analysis indicated that FLP-transduced piMEFs exhibited an increased % of cells in G1 phase and a deceased % in S/G2 phases, compared to that of the GFP-transduced piMEFs (**Fig. 4D**). It is noteworthy that the removal of SV40 T-antigen did not affect the expression of transcription factors Runx2 and Osterix (data not shown). Taken together, these results indicate that the proliferative activity of piMEFs can be effectively reversed by FLP recombinase

### Removal of SV40 T Antigen Reduces Osteogenic and Adipogenic Activities of piMEF Cells *In vitro*

**Figure 5:**
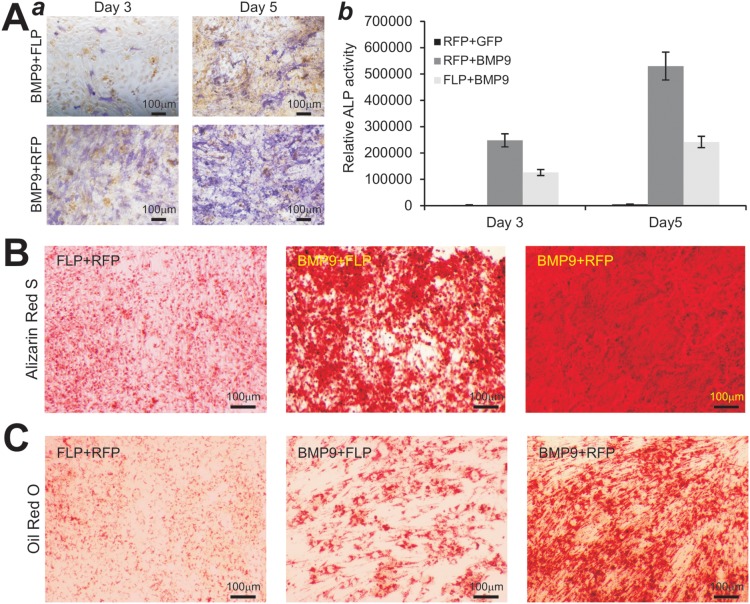
The removal of SV40 T antigen reduce the differentiation properties of the piMEFs. (**A**) Effect on ALP activity in piMEFs by FLP. AdFLP or AdGFP-transduced piMEFs were assessed histochemically (***a***) and quantitatively (***b***) at the indicated time points. (**B**) The effect of FLP-mediated reversal on late stage osteogenic differentiation of the piMEFs. Subconfluent piMEFs were infected with AdFLP or AdGFP and maintained in mineralization medium for 14 days. Cells were stained with Alizarin Red S. (**C**) The effect of FLP-mediated reversal on late stage adipogenic differentiation of the piMEFs. Subconfluent piMEFs were infected with AdFLP or AdGFP and maintained for 14 days. Cells were fixed and stained with Oil Red-O. All assay conditions were done in duplicate. Representative results are shown.

We analyzed if the differentiation potential of piMEFs was affected by FLP recombinase-mediated removal of SV40 T antigen. When piMEFs were co-infected with AdBMP9 and AdFLP or AdRFP, the ALP activity was decreased in FLP-transduced piMEFs, compared to that of RFP-infected control groups (**Fig. 5A**). We also determined the effect of FLP-mediated removal of T antigen on late stage differentiation of the piMEFs, and found that FLP-mediated removal of SV40 T antigen significantly reduced the BMP9-induced matrix mineralization, as assessed by Alizarin Red S staining, compared to that of the RFP-transduced piMEFs (**Fig. 5B**). Lastly, when subconfluent piMEFs were co-infected with AdBMP9 and AdFLP or AdRFP for 14 days and stained with Oil Red-O staining, we found that BMP9-induced adiogenic differentiation was reduced in FLP-transduced piMEFs, compared with that of RFP-transduced control piMEFs (**Fig. 5C**). Thus, these results indicate that FLP-tranduced piMEFs are still able to differentiate into osteogenic and adipogenic lineages, but significantly less effective than the RFP controls. One possibility is that FLP-mediated reversal of immortalization phenotype may reduce the differentiation potential of piMEFs due to the decreased progenitor cell repopulation.

### piMEFs can Effectively Induce Ectopic Bone Formation, Chondrogenesis and Adipogenesis upon BMP9 Stimulation *In vivo*

**Figure 6:**
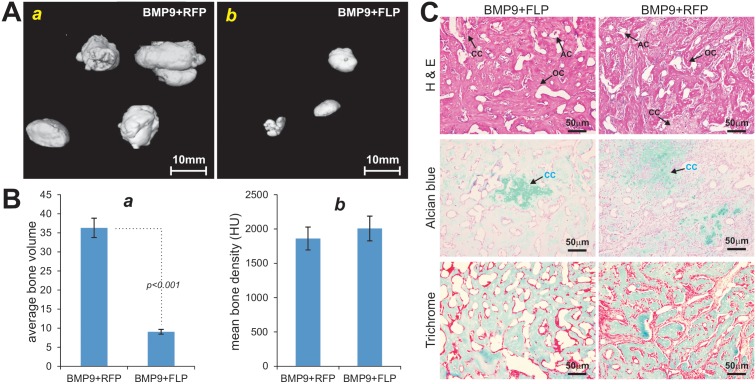
The piMEFs can effectively undergo ostepgenic, chondrogenic and adipogenic differentiation upon BMP9 stimulation *in vivo*. (**A**) The piMEFs without the removal of SV40 T Ag form larger ectopic bone masses. Subconfluent piMEFs were co-infected with BMP9, GFP and/or FLP for 16 h. The same number of cells were collected and injected into the flanks of athymic mice subcutaneously. Bony masses were retrieved from mice after 4 wk and subjected to µCT imaging. The scanning data were analyzed with Amira 5.3 software to obtain 3D iso-surface images. No masses were formed in the cells transduced with AdGFP or AdFLP alone. Representative results are shown. (**B**) The volumetric and relative density data of the ectopic bone masses were analyzed using the software Amira 5.3. HU, Hounsfield unit. (**C**) H & E, Alcian Blue, and Trichrome staining. The retrieved masses were fixed, decalcified, and subjected to H & E, Alcian Blue, and Trichrome staining. AC, adipocyte; CC, chondrocyte; OC, osteocyte.

We tested if piMEF cells retain multi-potency and are capable of differentiating into multiple lineages *in vivo*. Using the well-established stem cell implantation assay [32], [33], [35], [40]–[42], we co-infected piMEFs with AdBMP9 and AdFLP or AdRFP in culture and injected the transduced cells subcutaneously into athymic nude mice. Bony masses were retrieved from mice after 4 weeks and subjected to µCT imaging (**Fig. 6A**), while no masses were formed in the cells transduced with AdRFP or AdFLP alone (data not shown). In fact, piMEFs cells transduced with AdGFP or untreated piMEFs disappeared in 7–10 days after subcutaneous or intramuscular injection in athymic nude mice (data not shown), consistent with our previous observation with primary MEFs or iMEFs [58].

Consistent with our *in vitro* results, the FLP-mediated removal of SV40 T antigen from piMEFs led to the formation of smaller masses than that of GFP controls (**Fig. 6A**). Further analysis of the µCT data revealed that the average bone volume in BMP9-stimulated and FLP-transduced piMEFs was significantly smaller than that of the BMP9-stimulated and RFP-transduced piMEFs (*p<0.001*) (**Fig. 6B**, panel a). However, the relative mean bone density (measured in Hounsfield unit) upon BMP9 stimulation exhibited no significant difference between the FLP-transduced and RFP-transduced piMEFs (**Fig. 6B**, panel b). H & E staining of the retrieved masses indicate that both FLP-transduced piMEFs and RFP-infected piMEFs formed mature and fully mineralized osteoid matrix (**Fig. 6C**, top panel) while the presence of chondrocytes and adipocytes was readily observed. The presence of chondrocytes and chondroid matrix was further confirmed in both groups with Alcian Blue staining (**Fig. 6C** **,** middle panel). Masson Trichrome staining confirmed that both mineralized osteoid matrix and osteoid matrix were presented in FLP-treated and RFP-treated samples (**Fig. 6C**, bottom panel).

These *in vivo* results strongly suggest that piMEFs may give rise to osteogenic, chondrogenic and adipogenic lineages. The immortalization of piMEFs can be reversed by FLP recombinase, which may diminish the proliferative activity and hence differentiation potential. It is thus conceivable that these cells may be used as a model system for MSC biology and differentiation studies.

## Discussion

MEFs have attracted a lot attention for their potential role in elucidating stem differentiation, promoting tissue engineering, and functioning as gene vectors and immunomodulators [1]–[6], [11]–[15], [60], [61]. While bone marrow stromal was originally identified as the primary source, MSCs have been isolated from almost every type of tissue, including periosteum, brain, liver, bone marrow, adipose, skeletal muscle, amniotic fluid and hair follicle [62]–[71]. While MSCs isolated from various tissues share many similar characteristics, they exhibit significant differences in their expression profile and differentiation potential [72].

Primary MEFs have become a popular source of MSCss. However, the life span of primary MEFs is limited, and isolation of primary MEFs is time-consuming and labor-intensive. Thus, there is an unmet need to develop MEFs with permanent growth features. While the classical BALB/3T3 protocol proved the principle of cell immortalization [16], this approach is not efficient and thus often replaced by overexpression of oncogenes and/or inactivation of tumor suppressor genes [17]. In our studies, we employed SV40 T antigen, one of the most commonly used immortalization genes. The large T antigen encoded by SV40 plays essential roles in the infection of permissive cells, leading to production of progeny SV40 virions, and in the infection of nonpermissive cells, leading to malignant transformation [18], [19]. The ability of SV40 large T antigen to immortalize MEFs is largely dependent on its ability to complex with p53 [20].

Many other genes have been used to immortalize primary cells. The commonly used oncogenes may include telomerase (TERT), Kras, c-Myc, CDK4, cyclin D1, Bmi-1, and HPV 16 E6/E7, while the frequently inactivated tumor suppressor genes are p53, Rb, and p16^INK^, etc. Here, we demonstrate that the SV40 T antigen can effectively immortalize MEFs and the resulting piMEFs can be reversed by FLP-mediated removal of the SV40 T antigen. More importantly, our results strongly suggest that piMEFs may retain long-term proliferative activity and yet give rise to osteogenic, chondrogenic and adipogenic lineages upon BMP9 stimulation.

In this study, we demonstrate that *piggyBac* transposon-mediated stable expression of SV40 T antigen is an efficient approach to the immortalization of primary cells. Using a retroviral vector-based reversible immortalization system expressing SV40 T antigen [21], we previously immortalized several types of progenitors, including MEFs, mouse hepatic progenitor cells, mouse cardiomyogenic progenitor cells, and mouse melanoblastic progenitor cells [22]–[27]. However, the immortalization efficiency was relatively low because of the low retrovirus titers associated with the large cargo size for packaging.

The *piggyBac* transposon system offers significant advantages over the retroviral system [28], [73]. First, *piggyBac* vector can deliver large cargo sizes, up to 100 kb of DNA fragments, into mammalian cells. Second, unlike retroviral infection, *piggBac* vectors can be delivered into cells with multiple copies so it is easy to achieve high levels of transgene expression. Third, liposome-based transfection is more efficient than retroviral vector-mediated infection in vitro. Fourth, piggyBac exhibits non-random integration site selectivity and has a higher preference for integrations in regions surrounding transcriptional start sites [31]. Lastly, it is conceivable that piggyBac transposon can be removed from the host genome by its transposase and thus leaves no footprints. We are unable to remove the *piggyBac* vector from the piMEF genome because the wildtype transposase catalyzes the integration and excision of the transposon elements with equal efficiency. However, the excision-only/dominant forms of mutant *piggyBac* transposase have recently been reported [74], [75]. Thus, it is conceivable that the piggyBac transposon-mediated immortalization of primary cells can be reversible and footprintless.

In summary, we investigate if piggyBac transposon-mediated expression of SV40 T antigen can effectively immortalize MEFs without comprising the multi-potent properties of MEFs. Using the engineered piggyBac vector pMPH86 that contains the hygromycin and SV40 T antigen expression cassettes flanked with FRT sites. Our results show that MEFs can be effectively immortalized with SV40 T antigen, and the proliferative activity of the piMEFs can be effectively reversed by FLP recombinase. The piMEFs express MSC markers and can undergo osteogenic, chondrogenic, and adipogenic differentiation upon BMP9 stimulation both in vitro and in vivo. Taken together, our results have demonstrated that the reversibly immortalized piMEFs not only maintain long-term cell proliferation but also retain the ability to differentiate into multiple lineages. Thus, the reported *piggyBac* transposon-mediated expression of SV40 T immortalization system should be used as an effective tool to establish stable cells from primary progenitors isolated from limited tissue sources, which would be critical for basic and translational studies.
